# Potent Antioxidant and Anti-Tyrosinase Activity of Butein and Homobutein Probed by Molecular Kinetic and Mechanistic Studies †

**DOI:** 10.3390/antiox12091763

**Published:** 2023-09-14

**Authors:** Wenkai Pan, Ilaria Giovanardi, Tomiris Sagynova, Alice Cariola, Veronica Bresciani, Matteo Masetti, Luca Valgimigli

**Affiliations:** 1 Department of Chemistry “G. Ciamician”, University of Bologna, Via P. Gobetti 85, 40129 Bologna, Italy; 2 Tecnopolo di Rimini, Via Dario Campana 71, 47922 Rimini, Italy; 3 Department of Pharmacy and Biotechnology, University of Bologna, Via Belmeloro 6, 40126 Bologna, Italy; 4 Computational and Chemical Biology, Italian Institute of Technology, Via Enrico Melen 83, 16152 Genova, Italy

**Keywords:** skin whitening, food safety, chalcones, polyphenols, peroxyl radicals, melanin, kinetics, mechanism, molecular docking

## Abstract

Butein (BU) and homobutein (HB) are bioactive polyhydroxylated chalcones widespread in dietary plants, whose antioxidant properties require mechanistic definition. They were investigated by inhibited autoxidation kinetic studies of methyl linoleate in Triton™ X-100 micelles at pH 7.4, 37 °C. Butein had *k*_inh_ = (3.0 ± 0.9) × 10^4^ M^−1^s^−1^ showing a chain-breaking mechanism with higher antioxidant activity than reference α-tocopherol (*k*_inh_ = (2.2 ± 0.6) × 10^4^ M^−1^s^−1^), particularly concerning the stoichiometry or peroxyl radical trapping *n* = 3.7 ± 1.1 vs. 2.0 for tocopherol. Homobutein had *k*_inh_ = (2.8 ± 0.9) × 10^3^ M^−1^s^−1^, pairing the relative BDE_OH_ measured by radical equilibration EPR as 78.4 ± 0.2 kcal/mol for BU and estimated as 82.6 kcal/mol for HB. The inhibition of mushroom tyrosinase (mTYR) by HB and BU was also investigated. BU gives a reversible uncompetitive inhibition of monophenolase reaction with *K*_I_′ = 9.95 ± 2.69 µM and mixed-type diphenolase inhibition with *K*_I_ = 3.30 ± 0.75 µM and *K*_I_′ = 18.75 ± 5.15 µM, while HB was nearly competitive toward both mono- and diphenolase with respective *K*_I_ of 2.76 ± 0.70 µM and 2.50 ± 1.56 µM. IC_50_ values (monophenolase/diphenolase at 1 mM substrate) were 10.88 ± 2.19 µM/15.20 ± 1.25 µM, 14.78 ± 1.05 µM/12.36 ± 2.00 µM, and 33.14 ± 5.03 µM/18.27 ± 3.42 µM, respectively, for BU, HB, and reference kojic acid. Molecular docking studies confirmed the mechanism. Results indicate very potent antioxidant activity for BU and potent anti-tyrosinase activity for both chalcones, which is discussed in relation to bioactivity toward protection from skin disorders and food oxidative spoilage.

## 1. Introduction

**Figure 1:**
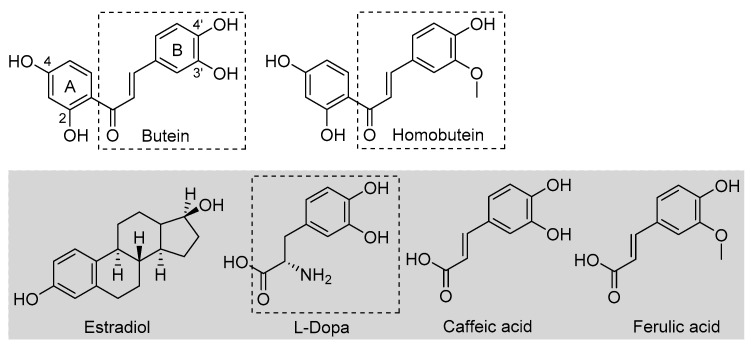
Structures of butein and homobutein, along with related reference compounds.

Butein is a natural polyphenolic chalcone (Figure 1) found in a very large variety of botanical sources belonging to different families, such as *Asteraceae* (e.g., *Coreopsis lanceolata* L., *Dahlia variabilis* Desf.), *Asparagaceae* (e.g., *Sansevieria liberica* Ger.), *Anacardiaceae* (e.g., *Semecarpus anacardium* L.), *Fabaceae* (e.g., *Butea frondosa* Roxb., *Butea monosperma* Taub., *Acacia pycnatha* Benth.), *Pinaceae* (e.g., *Abies pindrow* Royle.), *Solanaceae* (e.g., *Solanum lycopersicum* Lam.), and others [1]. As such, it is a very important dietary polyphenol that is specifically considered a nutraceutical owing to its many beneficial properties, which include protection against some forms of cancer and anti-angiogenic, anti-inflammatory, antidiabetic, neuroprotective, hepatoprotective, nephroprotective, and anti-hypertensive properties [1,2,3,4,5]. Plant extracts rich in butein (e.g., *D. variabilis*, *B. monosperma*, etc.) have a long tradition of use in folk medicine, particularly in China, Korea, and Japan [1,2]. Some identified mechanisms as the basis of its bioactivity include the up- or downregulation of enzymes, such as protein kinases, and interference with the NF-kB signaling pathway [1,2,3,4]. Structural similarity to estrogens (Figure 1) is attributed a role in its bioactivity [2]. However, bioactivity has also been associated with its antioxidant activity [6]. This has been the subject of different studies, highlighting an *indirect* antioxidant behavior via the activation of endogenous cellular antioxidant defenses [7]. It was recently demonstrated that a distinct mechanism to explain such antioxidant activity by butein is via the activation of the NRf2 signaling pathway [8].

An early study also indicated that butein has a direct antioxidant action: it can trap peroxyl radicals in water but not in hexane, and it was able to reduce the markers of oxidative damage (TBARS) in metal-catalyzed oxidation of LDL—an activity that was attributed to its ability in chelating transition metal ions, iron, and copper, preventing peroxidation initiated by Fenton reaction [9]. This would be a *preventive* albeit *direct* mechanism. Indeed, a recent computational study also pointed toward iron chelation to explain the activity of butein, which was surpassed by its analogue homobutein [10], differing only for methylation of the -OH in 3′ position in B-ring (Figure 1). Not much else is known about the antioxidant activity of homobutein, which also showed anti-cancer and anti-inflammatory activity [11], except it was found ineffective, at variance with butein, in reducing the production of reactive oxygen species (ROS) in breast cancer cells [6].

Butein and homobutein bear structural similarities to the well-established antioxidants caffeic acid and ferulic acid (Figure 1), which are known to inhibit lipid peroxidation by the direct *chain-breaking* mechanism by trapping chain-carrying alkylperoxyl radicals [12,13]. We hypothesized that the overlooked *chain-breaking* mechanism might also be prevalent in explaining the direct antioxidant behavior of butein and homobutein, which we investigated in detail on kinetic grounds by electron paramagnetic resonance (EPR) and by oxygen uptake kinetic studies in the inhibited peroxidation of methyl linoleate micelles.

One additional distinctive antioxidant action is via the inhibition of tyrosinase. Tyrosinase (EC 1.14.18.1) is a polyphenol oxidase enzyme, highly conserved in most living organisms, which regulates the biosynthesis of melanin, catalyzing two consecutive reactions: the monophenolase, consisting of the hydroxylation of a phenolic substrate (e.g., l-tyrosine) to the catechol, and the diphenolase, consisting in the oxidation of a catechol (e.g., l-dopa) to the *ortho*-quinone, which will then undergo further spontaneous oxidation and polymerization to afford the melanic pigments.

Inhibition of tyrosinase has major roles and applications, e.g., in biomedicine, to contrast skin pigmentation disorders like melasma [14,15,16,17] and in food safety, to prevent enzymatic food oxidative spoilage on storage [16,17,18,19].

The structural similarities of butein and homobutein with natural tyrosinase substrate l-dopa (Figure 1) suggest their possible bioactivity as inhibitors. Indeed, polyhydroxylated chalcones are known to inhibit tyrosinase [20,21,22], and some components of this class, such as morachalcone A found in *Morus alba*, are among the most potent natural tyrosinase inhibitors known to date [23]. No data are available in this regard for homobutein, while butein was reported ineffective toward diphenolase reaction, and data on activity toward monophenolase reaction appear conflicting [24]. Given their importance, we performed a detailed kinetic investigation on the inhibition of both tyrosinase reactions by butein and homobutein and used molecular docking computations to help rationalize the mechanism.

We anticipate that butein is an excellent chain-breaking antioxidant that largely outperforms homobutein and, beyond expectations, it outperforms even reference α-tocopherol (α-TOH) with an unusual mechanism. On the other hand, both butein and homobutein are potent inhibitors of both monophenolase and diphenolase tyrosinase reactions, with homobutein slightly outperforming butein while showing a partly different mechanism despite the similar structure.

## 2. Materials and Methods

### 2.1. Materials

Butein ((*E*)-1-(2,4-dihydroxyphenyl)-3-(3,4-dihydroxyphenyl)prop-2-en-1-one or 2′,3,4,4′-tetrahydroxychalcone) and homobutein ((*E*)-1-(2,4-dihydroxyphenyl)-3-(4-hydroxy-3-methoxyphenyl)prop-2-en-1-one or 2′,4,4′-trihydroxy-3-methoxychalcone) were purchased from Cymit Quimica (Barcelona, Spain). AAPH (2,2′-azobis(2-methylpropionamidine) dihydrochloride), methyl linoleate (MeLin; ≥98%) and Triton™ X-100, (*R,R,R*)-α-tocopherol (α-TOH), l-tyrosine (≥98%), l-dopa (3,4-dihydroxy-l-phenylalanine; ≥98%), kojic acid (5-hydroxy-2-hydroxymethyl-4*H*-4-pyranone; ≥98.5%), and mushroom tyrosinase (mTYR; EC 1.14.18.1, activity = 3410 units/mg) were obtained from Sigma-Aldrich (Milan, Italy) and used as received. Fresh mTYR stock solutions were prepared every second day and stored at 4 °C. Before use, tyrosinase activity was analyzed spectrophotometrically to define the necessary dilution to fixed tyrosinase Sigma units for consistent results. One Sigma unit corresponds to an increase in absorbance at 280 nm of 0.001 per minute at pH 6.8 in a 3 mL reaction mixture containing l-tyrosine. One Sigma unit corresponds to 1.65 × 10^−4^ international units (I.U.) for monophenolase activity and to 2.24 × 10^−2^ I.U. for diphenolase activity [25]. Di-*tert*-butylperoxide (Sigma-Aldrich) was percolated twice through activated basic alumina, and 2,4,6-tri-*tert*-butylphenol (TBP, 98%) was recrystallized from hexane. Stock solutions of AAPH phosphate buffer (pH 7.4) were prepared every day and stored at 4 °C between subsequent uses to avoid loss of title. Solvents and other chemicals were of the highest available grade (Sigma-Aldrich, Merck, VWR; Milan, Italy) and were used as received.

### 2.2. Inhibited Autoxidation Studies in Micelles

Autoxidation studies were performed as previously described [19,26], with slight modifications. In a typical experiment, 2.5 mL of air-saturated buffered (PBS, 50 mM, pH 7.4) aqueous dispersion of MeLin (final concentration 2.74 mM) in Triton™ X-100 (final concentration 16 mM) micelles were prepared by vortex mixing. A fresh stock solution of AAPH was added (final concentration 2.5 mM), immediately followed by addition (5–30 μL) of a stock solution of the antioxidant in acetonitrile (final concentration of 1–20 μM) and by additional vortex mixing (5 s). The mixture was sealed in a 2 mL glass vial provided with a PTFE-coated stirring bar and capped with the O_2_ sensor. The sample was equilibrated at 37 °C in a thermostatted bath equipped with a sealed magnetic stirrer, and oxygen concentration was monitored with time as previously described [19]. Oxygen consumption in the absence of antioxidants was compared with that recorded in the presence of butein, homobutein, or α-tocopherol (α-TOH) as the reference antioxidant [19]. The inhibition rate constant *k*_inh_ was obtained from oxygen consumption plots by Equation (1) for AH = butein or α-TOH and by Equation (2) for homobutein, where *R*_0_ and *R*_inh_ are the rates of O_2_ consumption in the absence and presence of the antioxidant [27,28,29], using *k*_p_ = 36 M^−1^s^−1^ and 2*k*_t_ = 3.52 × 10^5^ M^−1^s^−1^ for MeLin in micelles [30]. The stoichiometric factor *n* was determined from the length of the inhibited period τ by Equation (3) [29]. The rate of initiation *R*_i_ was determined in preliminary experiments using α-TOH as the inhibitor (*n* = 2) by Equation (3) [29].

(1) $$-\frac{d\left[O_{2}\right]}{dt}=\frac{k_{p}\left[RH\right]R_{i}}{nk_{inh}\left[AH\right]}+R_{i}$$

(2) $$\frac{R_{0}}{R_{inh}}-\frac{R_{inh}}{R_{0}}=\frac{nk_{inh}\left[AH\right]}{\sqrt{2k_{t}R_{i}}}\;$$

(3) $$n=\frac{R_{i}\tau }{\left[AH\right]}\;$$

### 2.3. Electron Paramagnetic Resonance (EPR) Spectroscopy

Deoxygenated acetonitrile solutions containing the phenols (0.01–0.001 M) and di-*tert*-butyl peroxide (10% *v*/*v*) were sealed under nitrogen in a 2 mm ID suprasil quartz EPR tube. The sample was inserted in the thermostatted (30 °C) cavity of an X-band EPR spectrometer and photolyzed with a mercury–xenon lamp (240–400 nm, max 4500 mW/cm^2^). Spectra were recorded with the following settings: modulation amplitude 0.2–1 Gauss, sweep width 30–60 Gauss, modulation frequency 100 kHz, frequency 9.76 GHz, sweep time 60 s, and microwave power 0.1–1 mW. Measured *g*-factors were corrected with respect to that of TEMPO (*g* = 2.0064) [31] and of DPPH radical [32]. In ReqEPR experiments, mixtures of TBP and butein were analyzed to obtain the molar ratio of the two equilibrating radicals by comparison of the digitized experimental spectra with computer-simulated ones, as previously described [33,34]. This afforded the equilibrium constant, *K*_eq_ [34]. Different irradiation power levels (20% to 100%) and different ratios of the two phenols were tested to guarantee that the two species were at equilibrium [33].

### 2.4. Tyrosnase Inbibition

The kinetics of tyrosinase reaction with or without inhibitor was studied by UV–Vis spectrophotometry, similar to previous methods [35,36,37], following our recent protocol [18]. Measurements were performed at 30 °C in phosphate buffer (50 mM, pH 6.8) in polystyrene low-volume cuvettes (1.5 mL, l = 1 cm) with a double-beam spectrophotometer. l-Tyrosine and l-dopa (5 levels, 0.05–1 mM) were used as the substrate of mushroom tyrosinase (mTYR, 5.0 U/mL and 2.5 U/mL, respectively, for mono- and diphenolase reactions) for monophenolase and diphenolase reactions, respectively. Butein or homobutein (0 to 12 μM) and kojic acid (0 to 50 μM) were comparatively tested as inhibitors. The concentrations 1.4 µM, 2.8 µM, 5.6 µM, and 11.2 µM were tested for butein (mono- and diphenolase inhibition) and for homobutein monophenolase inhibition, while the concentrations 0.175 µM, 0.7 µM, 1.4 µM, and 5.6 µM were used for diphenolase inhibition by homobutein. The reaction was monitored at 475 nm for up to 60 min, following dopachrome formation. Initial velocity (*V* = ΔA/Δmin) was converted in μM/min according to Lambert–Beer law using the molar extinction coefficient ε = 3700 M^−1^cm^−1^ for dopachrome at λ_max_ = 475 nm. Michaelis–Menten parameters (*K*_m_ e *V*_max_) were obtained by processing initial velocity vs. substrate concentration data by non-linear fitting to M-M Equation (4), using Sigmaplot 11.0 (Systat Software Inc., San Jose, CA, USA) [18]. Linearized Lineweaver–Burk Equation (5) was used to identify the inhibition mode [35]. In both equations, *V* indicates the measured initial rate of reaction, [S] is the initial substrate concentration, while *V*_max_ and *K*_m_ are, respectively, the maximum reaction rate (at saturating substrate concentration) and the M-M constant, with the substrate concentration yielding half-maximum rate [35].

(4) $$V=\frac{V_{\max}\;\left[S\right]}{K_{m}+\left[S\right]}$$

(5) $$\frac{1}{V}=\frac{K_{m}}{V_{\max}\;\left[S\right]}+\frac{1}{V_{\max}}$$

### 2.5. Stability of Inhibitors in the Presence of Oxygen

To evaluate whether butein and homobutein can be a substrate for mTYR and their stability toward oxidation under the experimental conditions of this study, they were incubated at 30 °C in air-saturated buffer (PBS, 50 mM, pH 6.8) solution at a concentration of 0.05 M and 0.1 M (by dilution of a concentrated stock in acetonitrile) in the presence or absence of mTYR (5 U/mL and 7.5 U/mL) and monitored over 60–90 min by recording the full UV–Vis spectrum (200–800 nm), and by recording the oxygen consumption in the O_2_ uptake apparatus described in Section 2.2 [18]. The kinetics of spectral variation or O_2_ consumption were analyzed using Sigmaplot 11.0.

### 2.6. Molecular Docking

Molecular docking calculations of the reversible inhibitors (butein and homobutein) and the mushroom tyrosinase structure were performed by using Autodock Vina 1.1.2 [36], Autodock v4.2.6 [37], and Autodock GPU [38]. The three-dimensional (3D) structure of the *Agaricus bisporus* tyrosinase (PDB ID: 2Y9X, Chain A) was downloaded from RCSB Protein Data Bank (https://www.rcsb.org (accessed on 3 February 2023)) as a deoxy form tyrosinase. The 2D structures of the compounds were drawn by ChemDraw Pro. 20.0 and converted to 3D structure by Chem3D Ultra 20.0 software. The AutoDockTools 1.5.6 [37] package was employed to generate the docking input files. All bound water and ligands of the protein were eliminated, and the polar hydrogen was added and optimized. The ligands were prepared by merging non-polar hydrogen atoms and defining rotatable bonds. All atoms within 3 Å from the center of mass of Histine in complex with the crystal structure were defined as a docking pocket using VMD 1.9.3 software. The search grid of the key site of tyrosinase was identified as center x: −7.645, center y: −25.444, and center z: −38.149 with dimensions size x: 25, size y: 25, and size z: 25. Considering the docking score for ligand-to-receptor binding, which involves electrostatic, van der Waals, and solvation energies, we chose the amino acid residues (VAL283, GLY281, ASN260, ARG268) as the flexible side chains to run the flexible docking [39]. In order to increase the docking accuracy, the value of exhaustiveness was set to 32, and the default parameters were used if it was not mentioned. Cu formal charge in the active pocket was set to +1. The best-scoring pose, as judged by the Vina docking score, was chosen and visually analyzed using PyMoL 2.5.0 software (http://www.pymol.org/, accessed on 5 December 2022). The root-mean-square deviation (RMSD) was validated between the co-crystallized heavy atoms coordinates of the inhibitors and the theoretical poses determined in the calculations using PyMoL 2.5.0 software. The interaction energies between the enzyme and inhibitors were then calculated using Autodock Vina 1.1.2, Autodock v4.2.6, and Autodock GPU.

### 2.7. Statistical Analysis

Each measurement was performed at least in triplicate and reported as average ± standard error. In autoxidation studies, 3–5 different concentrations were tested for each antioxidant. In tyrosinase kinetics, *V*_max_ and *V*_max_^app^ and *K*_m_ and *K*_m_^app^ in the absence and presence of inhibitors were determined from non-linear regression of M-M plots based on 5–14 concentrations of the substrate, each with 4 concentrations of the inhibitor, which were analyzed by Shapiro–Wilk test with significance set at *p* ≤ 0.05.

## 3. Results and Discussion

### 3.1. Antioxidant Activity in the Inhibited Autoxidation of Methyl Linoleate Micelles

To study the antioxidant activity of butein and homobutein on quantitative grounds, we performed inhibited autoxidation studies using biomimetic neutral micelles of methyl linoleate (MeLin) in aqueous Triton^™^ X-100 (with 50 mM PBS, pH 7.4, 37 °C), since it is a well-validated model we and others used in previous studies [19,26]. The reaction kinetics was followed by monitoring oxygen consumption via miniaturized NIR-fluorescence-quenching O_2_ probes, according to a previously validated protocol [19], both in the absence and in the presence of different concentrations of butein or homobutein. The reaction was initiated thermally in the aqueous phase by the controlled decomposition of the azo compound AAPH so as to clearly distinguish the chain-breaking mechanism from other efficacies based on interference with the initiation process (e.g., the Fenton reaction by metal chelation) and α-TOH was used as the reference antioxidant.

**Figure 2:**
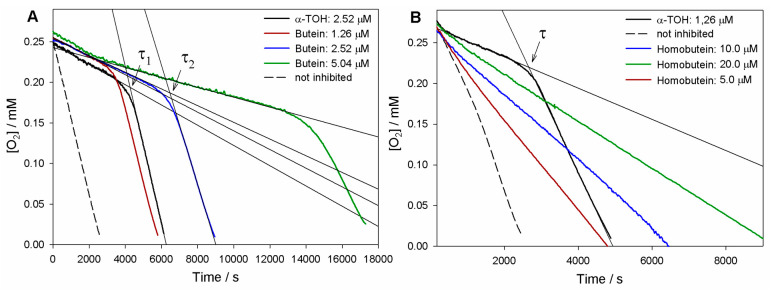
Oxygen uptake plots during the autoxidation of MeLin (2.74 mM) and Triton^™^ X-100 (16 mM) micelles in 50 mM PBS (pH 7.4), initiated by 2.5 mM AAPH at 37 °C and inhibited by (**A**) Butein at different concentrations or (**B**) Homobutein at different concentrations vs. α-TOH. In (**A**), τ_1_ and τ_2_ indicate, respectively, the inhibition time of 2.5 µM α-TOH and 2.5 µM butein.

As can be seen in Figure 2, both butein and homobutein effectively inhibited MeLin autoxidation, albeit with significantly different kinetic behavior. Reference α-TOH produced a marked inhibition of the autoxidation for a duration τ until it was completely consumed, then the autoxidation restarted at an uninhibited rate. The length of the inhibited period τ depends on the concentration of the antioxidant and on the stoichiometric factor *n* of peroxyl radical trapping (Equation (3)), i.e., the number of radicals trapped by one antioxidant molecule. Compared to α-TOH, homobutein was unable to produce a neat inhibited period; however, it slowed down autoxidation in a marked and dose-dependent fashion already in the low micromolar concentration range. This implies a substantial *k*_inh_ value, albeit significantly lower than α-TOH, which was determined from the slope of oxygen consumption plots via Equation (2) as (2.8 ± 0.9) × 10^3^ M^−1^s^−1^ (Table 1). The corresponding *k*_inh_ value for α-TOH was determined as (2.2 ± 0.6) × 10^4^ M^−1^s^−1^, in good agreement with the previous literature in the same model system [19,26]; therefore, the value for homobutein was about one order of magnitude lower. However, the *k*_inh_ value for homobutein was similar to or higher than that previously reported for other well-established antioxidants in the same model systems, e.g., bakuchiol [19] and resveratrol [26], which stands for its relevance as an antioxidant.

Instead, the antioxidant activity recorded for butein was surprisingly high (Figure 2). Not only was a neat inhibition period produced, but this was markedly more extended than that produced by α-TOH at the same concentration. The resulting stoichiometric factor *n* showed some variability from experiment to experiment, but this was consistently and significantly higher than the canonical *n* = 2 of α-TOH and the vast majority of phenolic antioxidants, averaging at 3.7 peroxyl radicals trapped by one molecule of butein (Table 1).

In addition, the rate of oxygen consumption during the inhibited period was slightly lower than with α-TOH, implying a faster trapping of peroxyl radicals (Equation (1)). Indeed, the measured *k*_inh_ = (3.0 ± 0.9) × 10^4^ M^−1^s^−1^ was slightly higher than that of α-TOH, completing the picture that indicates an overall higher antioxidant performance of butein.

### 3.2. EPR Spectroscopy

**Figure 3:**
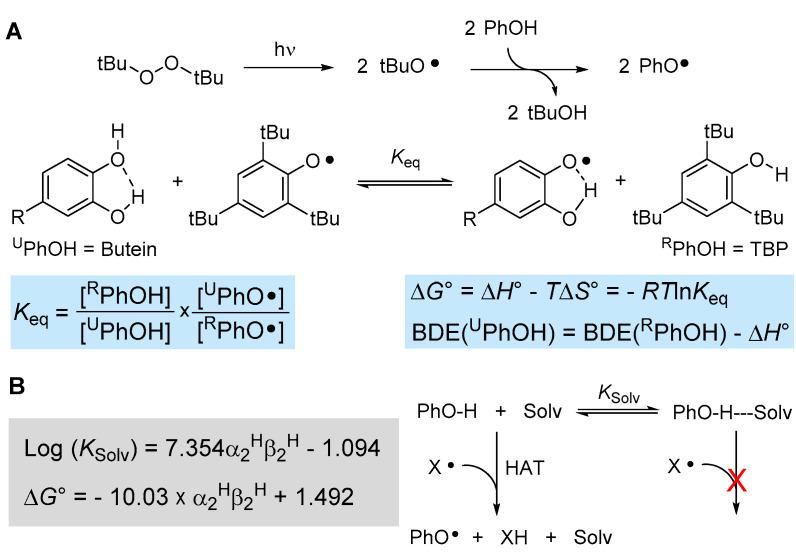
(**A**) ReqEPR equilibration study to measure the BDE of phenolic antioxidants by EPR spectroscopy and (**B**) influence of the solvent on the kinetics and thermodynamics of the HAT reaction.

The reactivity of (phenolic) chain-breaking antioxidants toward formal hydrogen atom transfer (HAT) to a radical such as peroxyl radicals is dictated by the strength of the phenolic O-H bond being broken, i.e., by its bond dissociation enthalpy (BDE_OH_), and by the steric hindrance in *ortho* position to the reactive OH [40,41]. Indeed, there are well-established Evans–Polanyi linear free-energy correlations between the BDE_OH_ and the inhibition rate constant *k*_inh_ for phenolic antioxidants [19,40]. One very accurate method to measure the BDE_OH_ is via radical equilibration experiments using electron paramagnetic resonance spectroscopy, the ReqEPR technique [40]. It consists of photolyzing the “unknown” phenol to be studied (^U^PhOH) in a mixture with a reference phenol (^R^PhOH) in the cavity of the EPR spectrometer with the addition of a peroxide (e.g., di-*tert*-butylperoxide) as a photochemical initiator. Analysis and deconvolution of the EPR spectrum containing both equilibrating radical species affords the equilibrium constant *K*_eq_ (see Figure 3), which in turn allows determining the ∆*G*° of equilibration. Since it has been shown that ∆*S*° is negligible for such an equilibrium [40], this affords the BDE_OH_ of ^U^PhOH if the value is known for ^R^PhOH (Figure 3A).

Using well-established reference 2,4,6-tri-*tert*-butylphenol (TBP, BDE_OH_ = 80.1 kcal/mol [40]), we performed the equilibration studies with butein in acetonitrile, owing to its insufficient solubility in apolar solvents, which are normally needed in these types of experiments to afford BDE values equivalent to the “gas-phase” [40]. The EPR spectra indicated that the phenoxyl radical of butein forms by HAT from the catechol B-ring (see Supplementary Materials) affording *K*_eq_ = 1.64 ± 0.63 and BDE_OH_ = 79.8 ± 0.2 kcal/mol (in acetonitrile at 298 K). To convert this value into a gas-phase equivalent, it is necessary to correct for the effect of the solvent, which increases the apparent BDE value by H-bonding to the phenols (see Figure 3B). Such solvent effects can be accounted for quantitatively, as detailed in Figure 3B, by using Abraham’s solvatochromic parameters α_2_^H^ and β_2_^H^, describing, respectively, the H-bond donating ability of the phenol and the H-bond accepting ability of the solvent [42,43]. Considering that the solvent effect is negligible for TBP due to steric hindrance by the *t*-butyl groups in *ortho* [40], that α_2_^H^ for catechol is 0.73 and β_2_^H^ for acetonitrile is 0.39 [42], the DBE measured in acetonitrile must be downscaled by −1.4 kcal/mol to afford BDE_OH_ = 78.4 ± 0.2 kcal/mol for butein in apolar solvent/gas-phase.

When we turned to homobutein, unfortunately, we were unable to obtain EPR spectra of sufficient quality to determine the BDE by ReqEPR, owing to the much lower persistency of the corresponding phenoxyl radical. However, its value can be estimated from that of butein considering the additive contribution of ring substituents on the BDE of phenols [40], i.e., −6 kcal/mol for *ortho*-OH and −1.8 kcal/mol for *ortho*-OCH_3_. This affords BDE_OH_ ~ 82.6 kcal/mol for homobutein.

The much lower BDE_OH_ of butein compared to homobutein justifies its much better antioxidant performance. Indeed, the BDE_OH_ of butein is lower than that of other catechol antioxidants such as hydroxytyrosol (80.8 kcal/mol [44]) and similar to well-established 3,5-di-*tert*-butylcatechol (78.2 kcal/mol [45]), which speaks for its excellent potential as a chain-breaking antioxidant.

### 3.3. Explaining the Excellent Antioxidant Activity of Butein in MeLin Micelles

While both butein and homobutein were effective antioxidants in the protection of methyl linoleate micelles, the performance of butein was truly exceptional and difficult to explain on the basis of the factors normally governing the reactivity of phenolic antioxidants. The value of *n* > 2 is hardly justified by the presence of other phenolic groups in the A ring as they are in relative *meta*-position and both conjugated with the electron-withdrawing carbonyl group, along with the occurrence of an intramolecular H-bond between the carbonyl and the OH in 2. This suggests a BDE_OH_ value > 86 kcal/mol for any OH group in A ring, which rules out their contribution in quenching peroxyl radicals [40]. While it cannot be excluded that the large *n* value arises from subsequent reactions of butein semiquinone radical to form dimeric structures endowed with radical trapping ability, as it was proposed for resveratrol [26], the concomitant very large *k*_inh_ value prompts a different explanation. Indeed, *k*_inh_ exceeds that of α-TOH despite butein having BDE_OH_ higher by 1.3 kcal/mol compared to α-TOH (see Section 3.2), which suggests the involvement of a different antioxidant mechanism. Likely, the catechol nature of butein allows its recycling during the autoxidation via the reduction of the semiquinone radical (QH^•^) and the quinone exhaust product (Q) by hydroperoxyl radicals (HOO^•^) released as a side reaction during the autoxidation of methyl linoleate [45,46,47], as depicted in Equations (6) and (7).

Q + HOO^•^ → QH^•^ + O_2_ (6)

QH^•^ + HOO^•^ → QH_2_ + O_2_ (7)

Reactions (6) and (7) have been found to be faster than the reaction of a catechol (QH_2_) with chain-carrying peroxyl radicals [48], which might help explain the higher reactivity of butein. This mechanism, based on the hydroperoxyl radical as a sacrificial reducing agent, has recently been demonstrated as key in explaining the antioxidant activity of melanin biopolymers (similarly based on the catechol/quinone redox chemistry) [48], and it is at the basis of the synergic antioxidant activity of catechols with terpenes like γ-terpinene [47]; furthermore, it explains the exceptional antioxidant activity of nitroxides in lipophilic environments such as in biological membranes [49]. We suggest it would have a role in the excellent antioxidant behavior of butein.

### 3.4. Kinetics of Inhibition of Mushroom Tyrosinase (mTYR)

**Figure 4:**
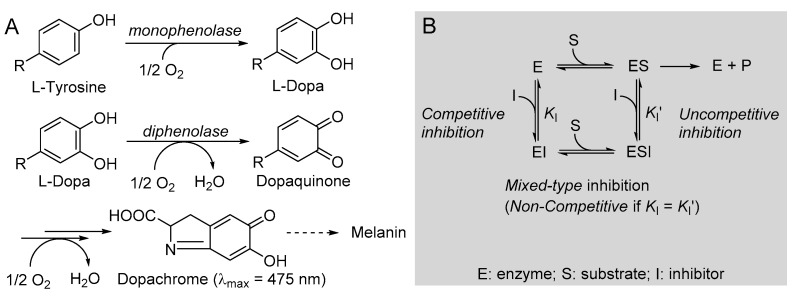
(**A**) Reactions involved in tyrosinase-catalyzed biosynthesis of melanin and (**B**) schematic representation of the possible reversible inhibition types.

The kinetics of monophenolase and diphenolase reactions of mTYR were investigated using, respectively, l-tyrosine and l-dopa as the natural substrates, in both cases monitoring the formation of dopachrome at λ_max_ = 475 nm, as depicted in Figure 4.

Non-linear regression of Michaelis–Menten (M-M) plot of the initial rate vs. substrate concentration (Equation (4)) afforded the parameters *V*_max_ = 3.85 ± 0.04 µM/min and the M-M constant *K*_m_ = 0.19 ± 0.01 mM for the monophenolase reaction and *V*_max_ = 11.69 ± 0.12 µM/min, *K*_m_ = 0.24 ± 0.01 mM for the diphenolase reaction, which are in good agreement with previous work from our group [18,19] and from others [38].

**Figure 5:**
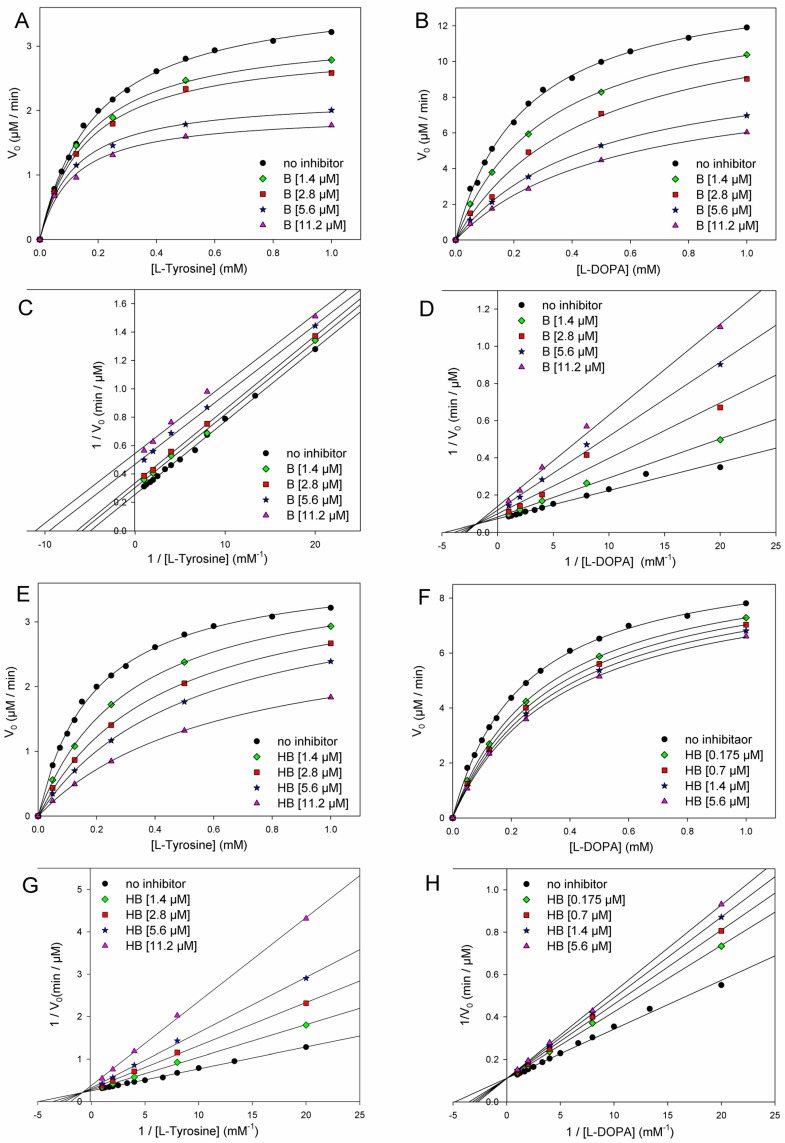
Kinetics of mTYR inhibition at 30 °C (pH = 6.8) by butein (**A**–**D**) and homobutein (**E**–**H**) showing the following: Michaelis–Menten plot of monophenolase activity (**A**,**E**) and of diphenolase activity (**B**,**F**) and the corresponding Lineweaver–Burk plots for monophenolase (**C**,**G**) and diphenolase (**D**,**H**) inhibition at the concentrations indicated in the legends. Enzyme concentrations for substrate l-dopa and l-tyrosine were 2.5 U/mL and 5.0 U/mL, respectively.

The addition of butein at micromolar levels significantly reduced the rate of dopachrome formation (Figure 5). In the monophenolase reaction (substrate = l-tyrosine), non-linear regression of the M-M plot showed a decrease of both the apparent *V*_max_ and *K*_m_ (*V*_max_^app^ and *K*_m_^app^) in the presence of increasing concentrations of the inhibitor (see Table 2), so that their ratio remained approximately constant. This infrequent kinetic behavior is typical of *uncompetitive* inhibitors, which act by binding the enzyme–substrate (E-S) complex rather than the free enzyme [35], as exemplified in Figure 4. Indeed, the Lineweaver–Burk (L-B) plot in Figure 5 showed parallel lines for the reaction without inhibitor or with the growing concentration of butein [35]. Inhibition potency can be accurately quantified by the M-M inhibition constant *K*_I_′, which represents the dissociation constant of the E-S-I complex—lower values indicate higher potency—that can be obtained by comparing *V*_max_ in the presence and absence of the inhibitor (Equation (8)) or *K*_m_ in the presence and absence of the inhibitor (Equation (9)) [35]. Results collected in Table 2 show good agreement between the two calculation methods, affording an averaged *K*_I_′ = 9.95 ± 2.69 µM, which indicates the high inhibition potency of butein toward the monophenolase reaction.

(8) $$V_{\max}^{\mathrm{app}}=\frac{V_{\max}}{1+\left[I\right]/K_{I}^{\prime }\;}\;\Rightarrow \;K_{I}^{\prime }=\frac{\left[I\right]}{V_{\max}/V_{\max}^{\mathrm{app}}-1}\;$$

(9) $$K_{m}^{\mathrm{app}}=\frac{K_{m}}{1+\left[I\right]/K_{I}^{\prime }\;}\;\Rightarrow \;K_{I}^{\prime }=\frac{\left[I\right]}{K_{m}/K_{m}^{\mathrm{app}}-1}\;$$

The behavior of butein toward the diphenolase reaction (substrate = l-dopa) was kinetically different, as indicated by the L-B plot in Figure 5, which shows the crossing of the regression lines for inhibited and uninhibited reactions in the second quadrant of the Cartesian plane. This is indicative of a mixed-type inhibition (competitive + uncompetitive) and is confirmed by data in Table 2, showing a decrease of *V*_max_ and a significant increase of *K*_m_ on increasing the inhibitor concentration [18,35]. It implies that butein is able to bind both the free enzyme E and the E-S complex with different affinities (Figure 2), as quantified by the respective dissociation constants *K*_I_ and *K*_I_′, which can conveniently be determined by Equations (10) and (11) [18,35].

(10) $$K_{I}=\left[I\right]/\alpha -1\;\mathrm{and}\;K_{I}^{\prime }=\left[I\right]/\alpha^{\prime }-1\;$$

where

(11) $$\alpha =(K_{m}^{\mathrm{app}}\;\times \;\alpha^{\prime })/K_{m}\;\mathrm{and}\;\alpha^{\prime }=V_{\max}/V_{\max}^{\mathrm{app}}$$

Results collected in Table 2 indicate that the competitive mechanism is largely prevailing since *K*_I_ = 3.30 ± 0.75 µM (competitive) is about 5-fold lower than *K*_I_′ (uncompetitive). Overall, kinetic data prove a high inhibition potency of butein both toward monophenolase and, particularly, toward diphenolase reactions of mTYR, at variance with previous knowledge (*vide infra*) [24].

Homobutein showed even higher inhibition efficacy toward both monophenolase and diphenolase reactions. Concerning the diphenolase reaction, clear inhibition was already detectable at a concentration as low as 175 nM (Figure 3). For both reactions, linearized L-B plots showed regression lines crossing close to the vertical axis, which would be indicative of competitive inhibition [18,19,35]. Nearly competitive inhibition is also confirmed by analysis of *V*_max_^app^ and *K*_m_^app^ data collected in Table 2: while *K*_m_ grows significantly on increasing the concentration of the inhibitor, *V*_max_ remains nearly constant. However, since the crossing points in L-B plots are slightly off-axis (in the II quarter), and some decrease in *V*_max_ is observed, at least for the highest inhibitor’s concentrations, we, more rigorously, treated both inhibitions as being of mixed-type and determined both M-M inhibition constants *K*_I_ and *K*_I_′ according to Equations (8) and (9). Results in Table 3 indicate that the competitive mechanism is largely prevailing as *K*_I_ values are one and two orders of magnitude lower than the corresponding *K*_I_′ for diphenolase and monophenolase reactions, respectively. This indicates that *K*_I_′ is poorly contributing to explaining the inhibiting behavior of homobutein. In other words, homobutein behaves as a nearly competitive inhibitor, i.e., it acts by competing with the natural substrate for interaction with the active site of the enzyme. Of interest, the low values of *K*_I,_ both for monophenolase and diphenolase reactions, as 2.75 ± 0.70 µM and 2.50 ± 1.56 µM, are indicative of high potency, surpassing both butein and reference kojic acid [18].

In a previous study from our group [18], kojic acid was found to give nearly competitive mixed-type inhibition toward the monophenolase reaction with *K*_I_ = 10.91 μM, while inhibition was mixed-type toward the diphenolase reaction, with *K*_I_ and *K*_I_′ values of 9.91 μM and 20.97 μM, respectively. These values are significantly higher than those found here for butein and particularly for homobutein, indicating substantially higher inhibition by the chalcones. Current values are at variance with a previous report on butein, which indicated competitive inhibition for the monophenolase reaction with *K*_I_ of 1.41 mM, i.e., orders of magnitude higher than found here and with a different mechanism [24]. The reasons for such diverse results are currently unknown.

### 3.5. Determination of IC_50_ against mTYR

Determination of M-M inhibition constants *K*_I_ and *K*_I_′, along with assignment of the inhibition mode, is the most reliable method to accurately quantify the potency of enzyme inhibitors, as it is independent of the experimental settings, like substrate and enzyme concentrations [18,35]. Nonetheless, it is most common in the scientific literature to find enzyme inhibitors characterized by their IC_50_; the concentration of the inhibitor is able to decrease the rate of the enzyme-catalyzed reaction, i.e., the apparent enzyme activity, by 50% under the same settings. Therefore, to allow comparison with literature data, we determined the IC_50_ both toward mono- and diphenolase reactions of mTYR for butein and homobutein, along with kojic acid chosen as reference inhibitor, using Langmuir isotherm Equation (12) [35], where at each substrate concentration, *V*_0_ and *V*_I_ indicate, respectively, the rate of reaction in the absence or in the presence of the inhibitor at concentration [I]. Results in Table 4 show a clear dependence on the substrate concentration, as expected [35]. While IC_50_ of butein decreases on increasing the concentration of l-tyrosine for monophenolase inhibition, owing to the uncompetitive mechanism that requires pre-formation of the E-S complex, values variably increase with the concentration of substrate for butein diphenolase inhibition and for homobutein and kojic acid, all showing a mixed-type inhibition with the (variable) prevalence of competitive behavior. This further highlights the limits of IC_50_ in quantifying inhibitors’ performance.

(12) $$\frac{V_{I}}{V_{0}}=\frac{1}{1+\frac{\left[I\right]}{IC_{50}}}$$

Nonetheless, potencies can be compared, taking [substrate] = 1 mM as “standard” settings [18]. Results for reference kojic acid are in good agreement with the previous literature [18,19,22,39], and IC_50_ for butein and homobutein, against both mono- and diphenolase reactions, were sensibly lower, confirming their higher inhibiting potency. Concerning butein, our values are lower but compatible with those of Khatib et al. [22] for monophenolase inhibition. However, they differ for diphenolase inhibition since butein was reported to be poorly effective even at concentrations > 100 µM [22]. While we do not have an explanation for this divergent outcome, we note that our IC_50_ values are fully consistent with the *K*_I_ values from kinetic studies.

### 3.6. Molecular Docking

To help rationalize the inhibiting mechanism of butein and homobutein, molecular docking simulations were performed against mTYR (PDBID: 2Y9X) to determine binding structures and affinities [16,50,51] using the AutoDock suite [39]. Validation of the docking settings was obtained with a 2Y9X crystal structure bearing co-crystallized tropolone in the active pocket. The tropolone inhibitor was removed and successfully re-docked in the original position.

Computations were first performed by individually docking butein, homobutein, and substrates l-tyrosine and l-dopa; setting flexibility in the ligand and rigid structure in the protein; and were repeated using AutoDock 4.2, AutoDock GPU, and AutoDock Vina to identify consistent binding poses and the protein residues which are mainly involved in interaction with the ligands. Results guided the setting of the flexible residues in AutoDock Vina, which was used for re-docking all the ligands so as to obtain optimized interaction at a low computational cost [37]. This removed unfavorable interactions and produced a significant decrease (i.e., numerical increase in the negative value) in the calculated binding energy.

**Figure 6:**
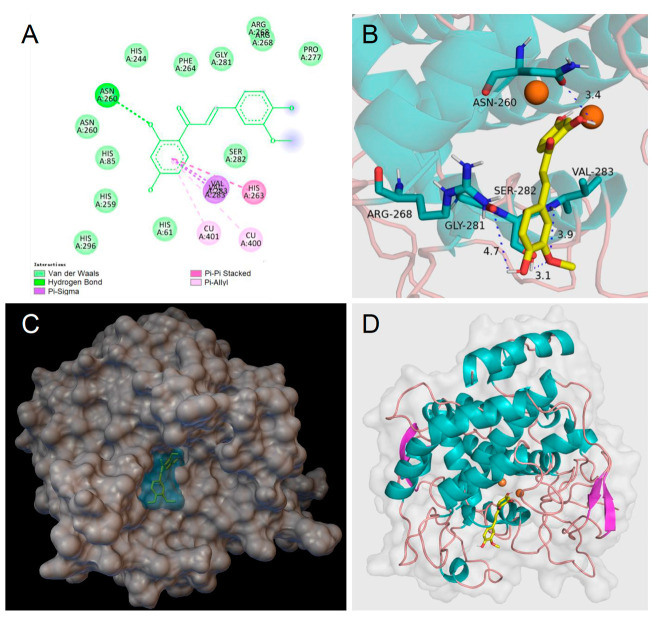
Molecular docking for homobutein (HB) to mTYR: (**A**) 2D interaction diagram of 2Y9X-HB complex, (**B**) detail of the active pocket with copper centers in orange, (**C**) molecular surface structure with the binding area in cyan, and (**D**) full view of 3D interaction of HB (in yellow) with 2Y9X.

**Figure 7:**
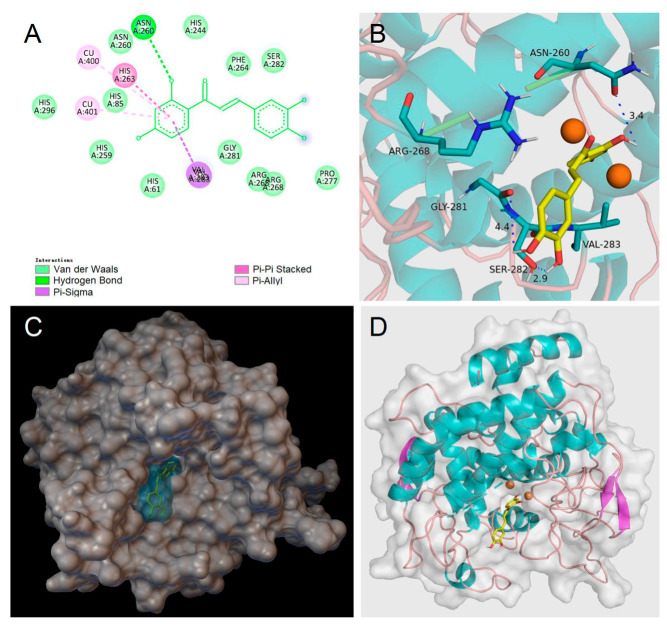
Molecular docking for butein (BU) to mTYR: (**A**) 2D interaction diagram of 2Y9X-BU complex, (**B**) detail of the active pocket with copper centers in orange, (**C**) molecular surface structure with the binding area in cyan, and (**D**) full view of 3D interaction of BU (in yellow) with 2Y9X.

Homobutein’s stabilizing interactions were mainly with ASN260, VAL283, VAL263, and HIS85 (Figure 6), and the binding energy was—7.14 kcal/mol, just slightly lower than calculated for butein (−6.86 Kcal/mol) stabilized by ARG268, ASN260 VAL283, and ALA286 (Figure 7). The lower binding energy for homobutein compared to butein matches with the lower *K*_I_ value for homobutein compared to butein measured for diphenolase inhibition (see Table 2 and Table 3), although calculated binding energies should not be overinterpreted, and differences in the order of 0.5 kcal/mol or lower should be regarded as merely indicative [51].

Of interest, both inhibitors had lower energies than the ligands, i.e., l-dopa, −6.31 kcal/mol, and l-tyrosine, −5.86 kcal/mol, which would justify their displacement and competitive inhibition. Further studies would be needed to help rationalize the uncompetitive inhibition of butein when the substrate is l-tyrosine, which we plan to pursue in further work. Meanwhile, current results lend support to our kinetic measurements, indicating prevailing competitive, reversible inhibition of mTYR by both butein and homobutein.

### 3.7. Stability of Butein and Homobutein toward Air and mTYR

Both butein and homobutein are polyphenols, mimicking the structure of natural l-tyrosine and l-dopa substrates; therefore, besides being potent inhibitors, they might also behave as alternative substrates of mushroom tyrosinase, being transformed by the enzyme during the kinetic studies, which would affect the measured kinetics. This has been previously reported for some phenolic inhibitors like caffeic acid and ferulic acid [52]. To investigate this aspect, both butein and homobutein were tested as substrates for mTYR, i.e., they were incubated in PBS at pH 6.8 (30 °C) in the presence and absence of (7 U/mL) mTYR at a much higher concentration than when used as inhibitors (0.05 mM and 0.1 mM, similar to those used for substrates l-tyrosine and l-dopa), in the absence of other substrates, i.e., without l-tyrosine and l-dopa. Their full UV–Vis spectrum was monitored for 60 min. Results showed a rapid spectral variation attributable to the complexation with the enzyme for butein, followed by a very slow decay of its concentration, which was identical in the presence or absence of the enzyme (see Supplementary Materials). Homobutein instead showed no initial spectral variation but a very slow decay of its concentration, which was identical in the presence and absence of the enzyme. This indicates that neither butein nor homobutein behave as substrates of mTYR. To confirm our judgment, we repeated the experiments by monitoring the oxygen consumption during the reactions, according to our previously validated protocol [18,19], since O_2_ is the obliged oxidant during the tyrosinase reaction and its time-course would parallel the consumption of substrates [18]. Both butein and homobutein showed negligible oxygen consumption with a rate of 2–4 nM/s (120–480 nM/min), i.e., orders of magnitude slower than that found for natural substrates l-tyrosine and l-dopa under similar settings [18,19]. Additionally, oxygen consumption was nearly identical in the absence and presence of the enzyme. Since this kinetic study does not rely on the formation of any specific oxidation product with a detectable spectrum and it is not affected by interference from other species absorbing in the same spectral window, it rules out that butein or homobutein are significant substrates for mTYR (see Supplementary Materials). Both UV–Vis and O_2_ uptake kinetics converge, showing some spontaneous slow oxidation of the chalcones in solution; however, the measured rates imply a negligible consumption during the typical experiment under our settings, ruling out any significant interference in the measured kinetics.

## 4. Conclusions

Both butein and homobutein are effective antioxidants working with a chain-breaking mechanism consisting of the fast trapping of chain-carrying peroxyl radicals (*k*_inh_ 3.0 × 10^4^ and 2.8 × 10^3^ M^−1^s^−1^, respectively). The lower BDE_OH_ of catechol butein (78.4 kcal/mol) compared to homobutein (82.6 kcal/mol) justifies its higher reactivity. However, it explains the outstanding antioxidant performance in the protection of methyl linoleate micelles only in part. Indeed, butein was able to outperform nature’s premiere antioxidant, α-tocopherol, despite its BDE_OH_ being higher by 1.3 kcal/mol, and, most interestingly, butein was able to trap almost twice as many peroxyl radicals per molecule of antioxidant compared to α-tocopherol. This behavior is reminiscent of the behavior previously found for catechol/quinone antioxidant systems [47] and, most notably, by catechol-based polydopamine [48] in the presence of a source of hydroperoxyl (HOO^•^) radicals. It is based on the very fast reduction by HOO^•^ of the semiquinone and quinone from the antioxidant to re-generate fresh catechol ready to carry on the inhibition. However, it was observed here without the deliberate addition of a source of hydroperoxyl radicals. We suggest that butein is able to exploit the HOO^•^ radical formed as a side event during the autoxidation of methyl linoleate [45,46,47]. This mechanism is likely of major relevance in biological systems, where O_2_^-•^/HOO^•^ is produced under both physiologic and pathologic conditions, which lends support to the redox bioactivity reported for butein in previous studies [6,7], complementing other mechanisms such as iron chelation [10] and induction of the NRf2 signaling system [8]. Indeed, catechol-type antioxidants have been shown to possess major potential in handling redox-related pathologies like neurological disorders [53].

Along with their antioxidant activity, both butein and homobutein have major efficacy in the inhibition of tyrosinase, showing a prevailing competitive mechanism when l-dopa is the substrate (diphenolase reaction), with *K*_I_ of 3.30 and 2.50 µM, respectively, both outperforming the reference kojic acid. This mechanism, which is supported by molecular docking studies, is investigated for the first time for homobutein and is at variance from previous knowledge for butein. Both chalcones were also active against the l-tyrosine substrate (monophenolase), albeit with a different mechanism, which was uncompetitive for butein (*K*_I_′ = 9.95 µM), and it was nearly competitive for homobutein (*K*_I_ = 2.76 µM). While the mechanism of homobutein is nicely supported by molecular docking, that of butein appears to require further efforts for full rationalization.

Of interest, the combined antioxidant and anti-tyrosinase activities open previously unexplored applications for the two chalcones, particularly in dermatology, to contrast photoaging, melasma, and age-related pigmentation disorders [14] and in food safety, where they would protect from oxidative food spoilage, both directly caused by oxygen and enzyme-mediated [19]. Clearly, such potential would require further studies, e.g., in cells or in food samples, for full validation and possible exploitation.

## Figures and Tables

**Table 1:** Inhibition rate constant, stoichiometric factor, and bond dissociation enthalpy of the reactive OH for the investigated chalcones vs. α-TOH in the AAPH (2.5 mM) initiated autoxidation of MeLin/Triton^™^ X-100 micelles at 37 °C, pH 7.4.

Antioxidant	*k*_inh_/10^3^ M^−1^s^−1^	*n*	BDE_OH_/kcal/mol
Butein	29.8 ± 9.2	3.7 ± 1.1	78.4 ± 0.2
Homobutein	2.8 ± 0.9	--	82.6 ^1^
α-TOH	22.4 ± 5.8	2 ^2^	77.1 ^3^

^1^ Estimated from the value for butein, considering the additive contribution of substituents (see Section 3.2). ^2^ Reference value. ^3^ From ref. [29].

**Table 2:** Kinetic parameters for the inhibition of mTYR by butein at 30 °C (pH 6.8).

**Inhibition of Monophenolase Reaction (Substrate = l-Tyrosine)**				
**Butein** **(µM)**	***K*_m_ or *K*_m_^app^ (mM)**	***V*_max_ or *V*_max_^app^ (µM/min)**	***K*_I_′ (µM)** ***Calc. from V_max_***	***K*_I_′ (µM)** ***Calc. from K_m_***
0	0.19 ± 0.01	3.85 ± 0.04	-	-
1.4	0.16 ± 0.02	3.24 ± 0.07	7.44	7.47
2.8	0.15 ± 0.01	3.02 ± 0.05	10.19	10.50
5.6	0.12 ± 0.02	2.21 ± 0.04	7.55	9.60
11.2	0.11 ± 0.01	1.95 ± 0.07	11.49	15.40
Average			9.17 ± 2.01	10.74 ± 3.36
**Inhibition of Diphenolase Reaction (Substrate = l-Dopa)**				
**Butein** **(µM)**	***K*_m_ or *K*_m_^app^ (mM)**	***V*_max_ or *V*_max_^app^ (µM/min)**	***K*_I_ (µM)** ***Competitive***	***K*_I_′ (µM)** ***Uncompetitive***
0	0.24 ± 0.01	11.78 ± 0.22	-	-
1.4	0.32 ± 0.01	10.93 ± 0.22	3.21	18.04
2.8	0.45 ± 0.06	10.60 ± 0.88	2.59	25.24
5.6	0.47 ± 0.02	8.17 ± 0.21	3.07	12.68
11.2	0.54 ± 0.03	7.42 ± 0.26	4.35	19.05
Average			3.30 ± 0.75	18.75 ± 5.15

**Table 3:** Kinetic parameters for the inhibition of mTYR by homobutein at 30 °C (pH 6.8).

**Inhibition of Monophenolase Reaction (Substrate = l-Tyrosine)**				
**Homobutein** **(µM)**	***K*_m_ or *K*_m_^app^ (mM)**	***V*_max_ or *V*_max_^app^ (µM/min)**	***K*_I_ (µM)** ***Competitive***	***K*_I_′ (µM)** ***Uncompetitive***
0	0.19 ± 0.01	3.85 ± 0.04	-	-
1.4	0.31 ± 0.01	3.83 ± 0.04	2.19	268.10
2.8	0.42 ± 0.02	3.78 ± 0.05	2.24	151.20
5.6	0.52 ± 0.02	3.63 ± 0.06	2.94	92.40
11.2	0.63 ± 0.01	3.15 ± 0.03	3.67	50.40
Average			2.76 ± 0.70	140.53 ± 94.57
**Inhibition of Diphenolase Reaction (Substrate = l-Dopa)**				
**Homobutein** **(µM)**	***K*_m_ or *K*_m_^app^ (mM)**	***V*_max_ or *V*_max_^app^ (µM/min)**	***K*_I_ (µM)** ***Competitive***	***K*_I_′ (µM)** ***Uncompetitive***
0	0.24 ± 0.01	11.60 ± 0.22	-	-
0.175	0.27 ± 0.01	11.48 ± 0.22	1.28	16.75
0.70	0.34 ± 0.05	11.30 ± 0.88	1.54	26.38
1.4	0.36 ± 0.02	11.10 ± 0.21	2.46	30.83
5.6	0.49 ± 0.08	10.84 ± 0.26	4.72	79.01
Average			2.50 ± 1.56	38.24 ± 27.81

**Table 4:** Values of IC_50_ for butein, homobutein, and kojic acid in the inhibition of mTYR (30 °C, pH 6.8) at each concentration of substrate (l-Tyrosine or l-Dopa) for mono- and diphenolase reactions.

	Butein IC_50_ (µM)		Homoutein IC_50_ (µM)		Kojic Acid IC_50_ (µM)	
Substrate (mM)	*Monophenolase*	*Diphenolase*	*Monophenolase*	*Diphenolase*	*Monophenolase*	*Diphenolase*
0.05	45.05 ± 18.31	3.76 ± 0.96	4.01 ± 0.66	3.04 ± 0.84	15.51 ± 3.52	4.81 ± 0.52
0.125	35.60 ± 28.42	4.12 ± 1.39	4.58 ± 0.88	3.83 ± 0.94	18.64 ± 3.85	8.23 ± 1.05
0.25	12.87 ± 3.22	5.38 ± 0.90	6.06 ± 0.95	5.60 ± 1.07	20.52 ± 2.71	9.31 ± 1.75
0.50	12.26 ± 2.52	9.31 ± 0.85	8.75 ± 1.18	7.57 ± 1.11	24.91 ± 2.92	12.03 ± 2.12
1.00	10.88 ± 2.19	15.20 ± 1.25	14.78 ± 1.05	12.36 ± 2.00	33.14 ± 5.03	18.27 ± 3.42

## Data Availability

Data are available within the article and Supplementary Materials.

## Supplementary Materials

The following supporting information can be downloaded at https://www.mdpi.com/article/10.3390/antiox12091763/s1. Figures S1 and S2: EPR spectra of butein and TPB mixtures; Figures S3–S10: Uv–Vis spectra of butein and homobutein incubated with mTYR and kinetic analysis; Figures S11 and S12: plots of O_2_ uptake during incubation of butein and homobutein with mTYR.
