# Supplementary material for: Potent Antioxidant and Anti-Tyrosinase Activity of Butein and Homobutein Probed by Molecular Kinetic and Mechanistic Studies
Source: Antioxidants (Basel). 2023 Sep 14;12(9):1763. doi: 10.3390/antiox12091763 (PMC10525132; doi:10.3390/antiox12091763)
Supplement: Supplementary file 1 [file antioxidants-12-01763-s001.zip › antioxidants-2586565-supplementary.pdf]

Potent Antioxidant and Anti-Tyrosinase activity of Butein and Homobutein probed by molecular kinetic and mechanistic studies

Wenkai Pan [1], Ilaria Giovanardi [1,2], Tomiris Sagynova [1,2], Alice Cariola [1,2], Veronica Bresciani [3], Matteo Masetti [3] and Luca Valgimigli [1,2,*]

Supplementary Materials

Table of contents

Figure S1. EPR spectrum of the mixture of TBP and butein 2.5:1 in acetonitrile P 2
Figure S2. EPR spectrum of the mixture of TBP and butein 1:8:1 in acetonitrile P 2
Figure S3. Variation of UV-Vis spectrum of 0.1 mM butein in air-saturated PBS with mTYR P 3
Figure S4. Variation of UV-Vis spectrum of 0.05 mM butein in air-saturated PBS with mTYR P 3
Figure S5. Variation of UV-Vis spectrum of 0.05 mM butein in air-saturated PBS without enzyme P 3
Figure S6. Variation of UV-Vis spectrum of 0.1 mM homobutein in air-saturated PBS with mTYR P 4
Figure S7. Variation of UV-Vis spectrum of 0.0.5 mM homobutein in air-saturated PBS with mTYR P 4
Figure S8. Variation of UV-Vis spectrum of 0.1 mM homobutein in air-saturated PBS without mTYR P 4
Figure S9. Kinetic analysis of butein variation monitored at 390 nm with and without mTYR P 5
Figure S10. Kinetic analysis of homobutein variation monitored at 3850 nm with and without mTYR P 5
Figure S11. Oxygen consumption upon incubation of butein 0.1 mM with and without mTYR P 6
Figure S12. Oxygen consumption upon incubation of homobutein 0.1 mM with and without mTYR P 7

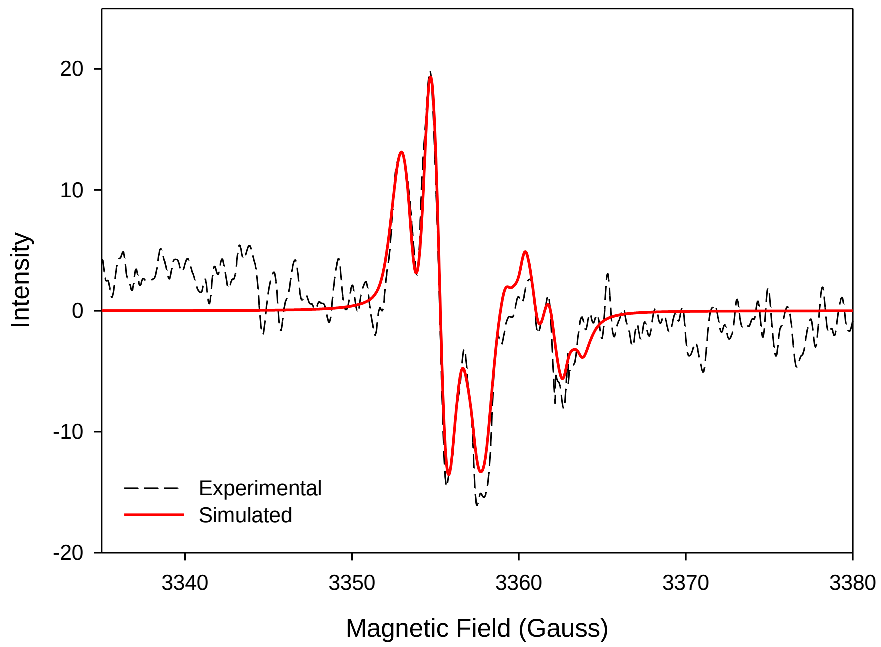

Figure S1. EPR spectrum (two scans) obtained upon UV irradiation of a mixture of 2,4,6-tri-*tert*-butylphenol (TBP) and butein in a ratio of 2.5:1 in acetonitrile containing 20% di-*tert*-butylperoxide, and its computer simulation for a radical ratio TBP:butein of 1.3:1. Simulation was obtained with the following parameters (in Gauss = 0.1 mT). TBP: a(2H) 1.78 G, a(27H) 0.12 G, LW 1.20 G, Lorenz. 40%, g = 2.0046; Butein: a(H) 1.58 G, a(H) 5.76 G, a(H) 1.02 G, a(H) 0.54 G, a(H) 1.22 G, a(H) 0.22 G, LW 0.61 G, Lorenz. 100%, g = 2.0027.

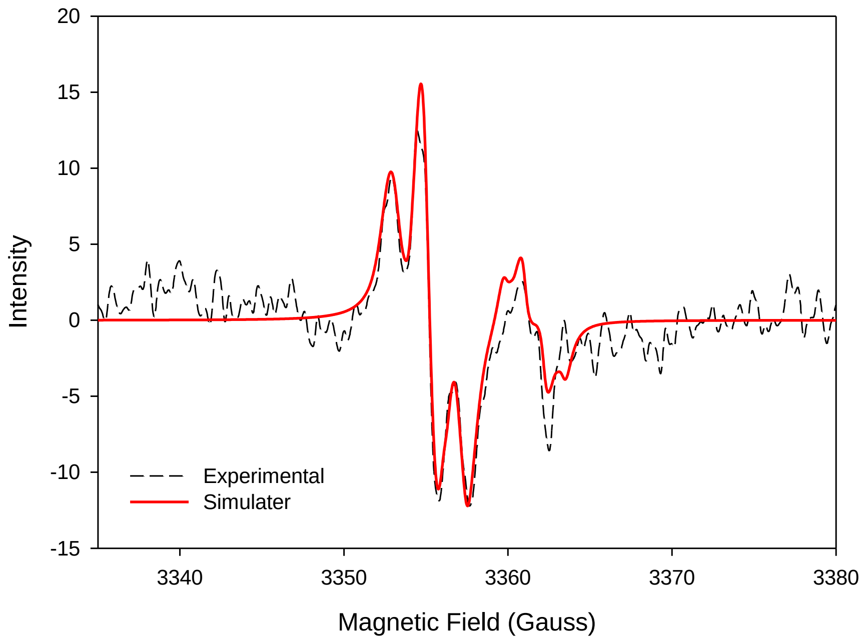

Figure S2. EPR spectrum (two scans) obtained upon UV irradiation of a mixture of 2,4,6-tri-*tert*-butylphenol (TBP) and butein in a ratio of 1.8:1 in acetonitrile containing 20% di-*tert*-butylperoxide, and its computer simulation for a radical ratio TBP:butein of 1:1.

Analysis of stability of butein and homobutein toward air-oxidation and mTYR catalyzed oxidation.

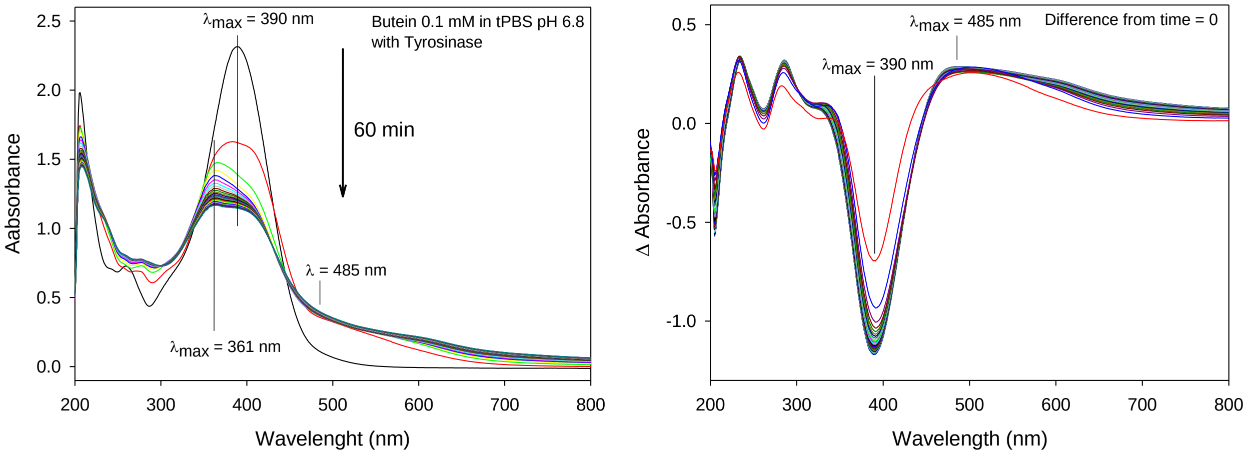

Figure S3. Variation of UV-Vis spectrum of 0.1 mM butein in air-saturated PBS pH 6.8 upon addition of 7 U/ml of mushroom tyrosinase at 30°C, showing both the full spectra (left) and the spectral difference from t=0 (right).

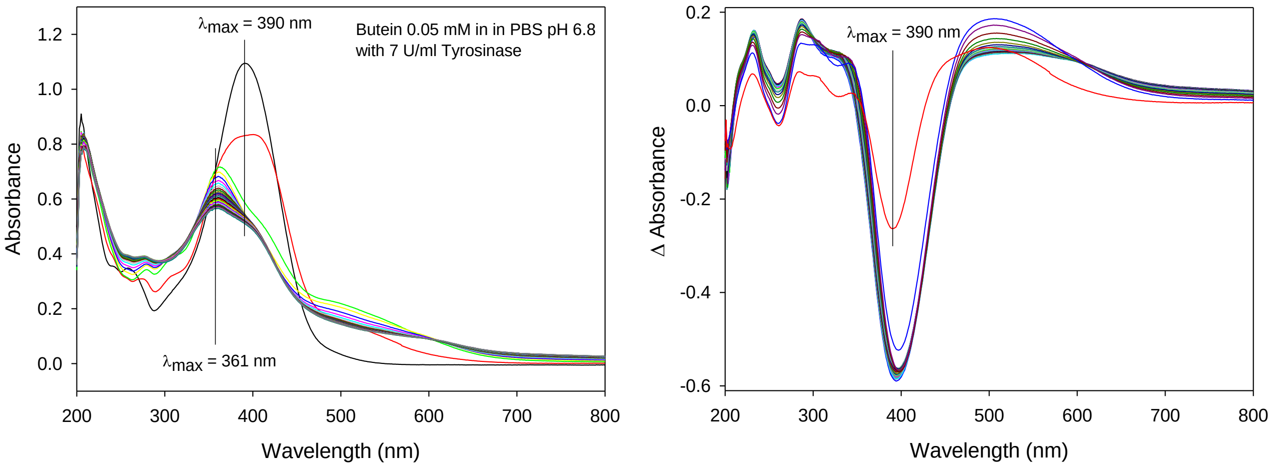

Figure S4. Variation of UV-Vis spectrum of 0.05 mM butein in air-saturated PBS pH 6.8 upon addition of 7 U/ml of mushroom tyrosinase at 30°C, showing both the full spectra (left) and the spectral difference from t=0 (right).

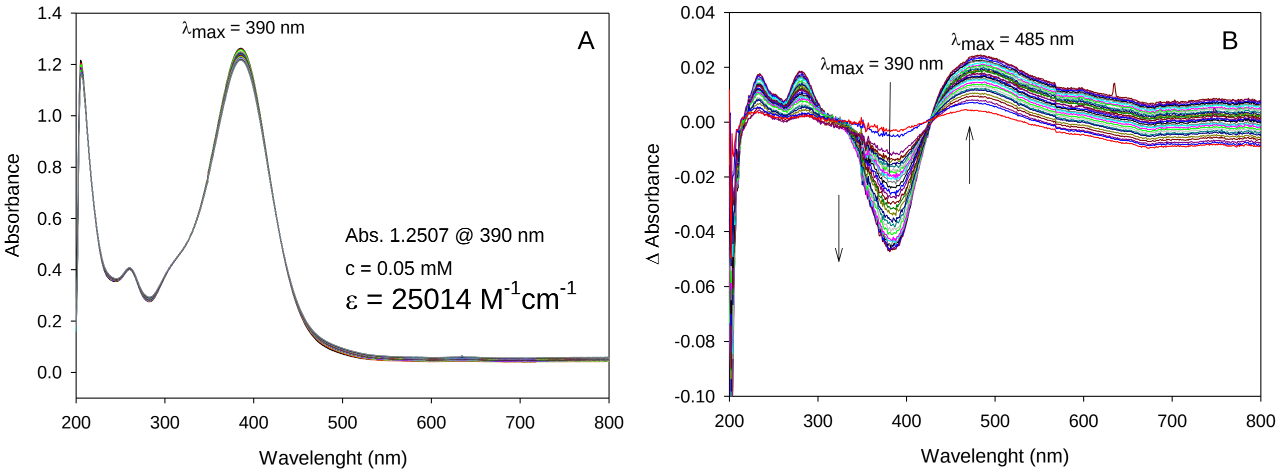

Figure S5. Variation of UV-Vis spectrum of 0.05 mM butein in air-saturated PBS pH 6.8 without enzyme at 30°C, showing both the full spectra (A) and the spectral difference from t=0 (B).

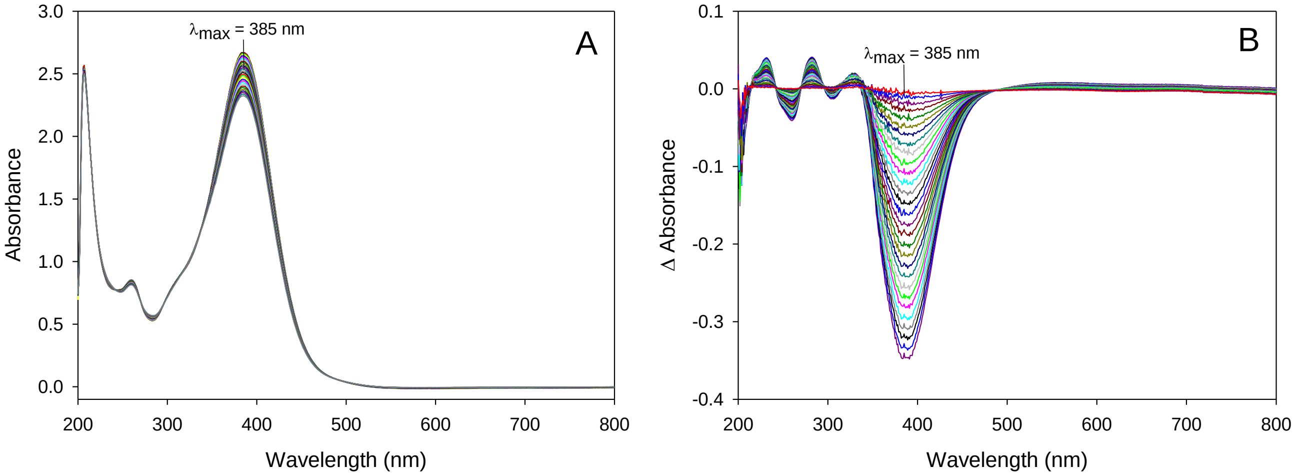

Figure S6. Variation of UV-Vis spectrum of 0.1 mM homobutein in air-saturated PBS pH 6.8 upon addition of 7 U/ml of mushroom tyrosinase at 30°C, showing both the full spectra (A) and the spectral difference from t=0 (B).

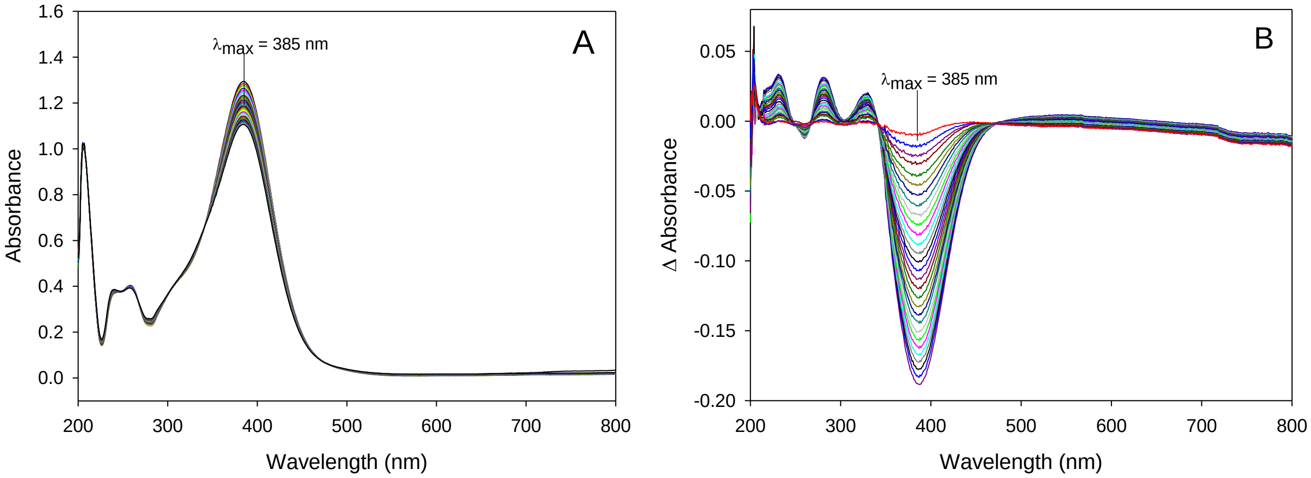

Figure S7. Variation of UV-Vis spectrum of 0.05 mM homobutein in air-saturated PBS pH 6.8 upon addition of 7 U/ml of mushroom tyrosinase at 30°C, showing both the full spectra (A) and the spectral difference from t=0 (B).

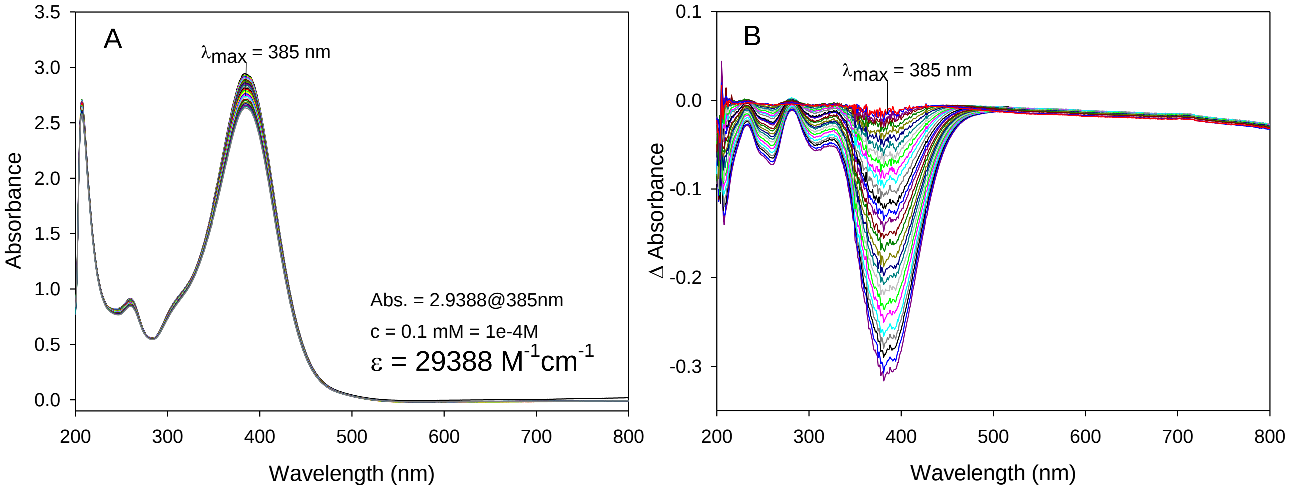

aaaa

Figure S8. Variation of UV-Vis spectrum of 0.1 mM homobutein in air-saturated PBS pH 6.8 without enzyme at 30°C, showing both the full spectra (A) and the spectral difference from t=0 (B).

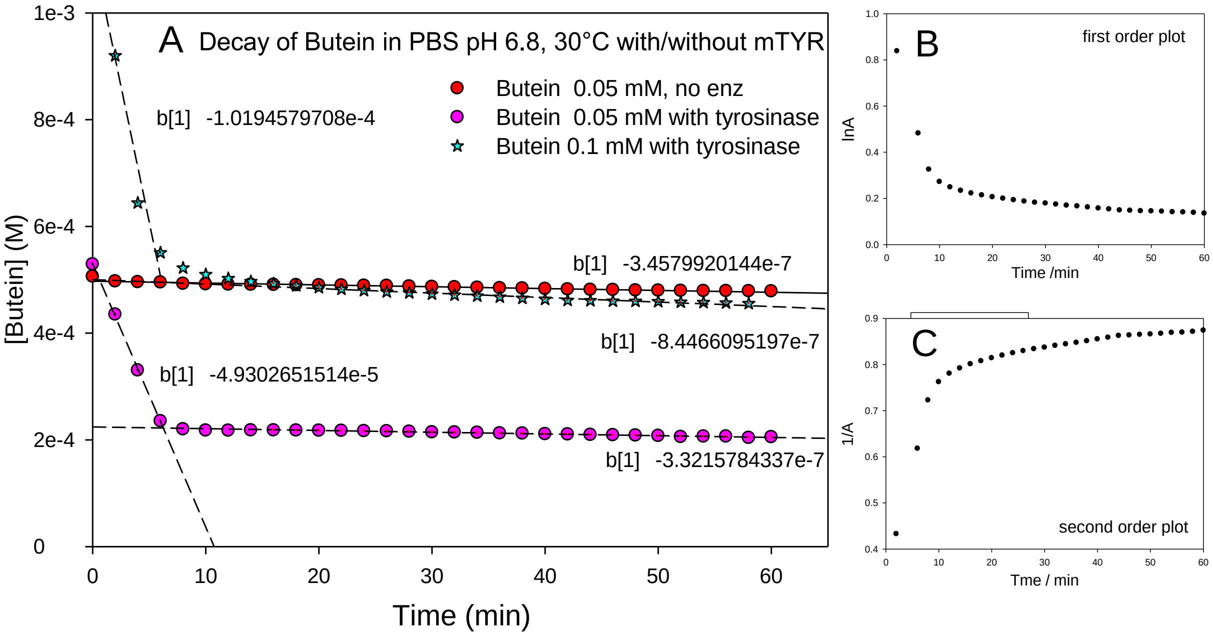

Figure S9. Kinetic analysis of the variation of butein concentration monitored at 390 nm in air-saturated PBS pH 6.8 at 30°C, in the presence and absence of mTYR, showing segmented zero-order fitting (A) first order plot (B) and second oerder plot (C).

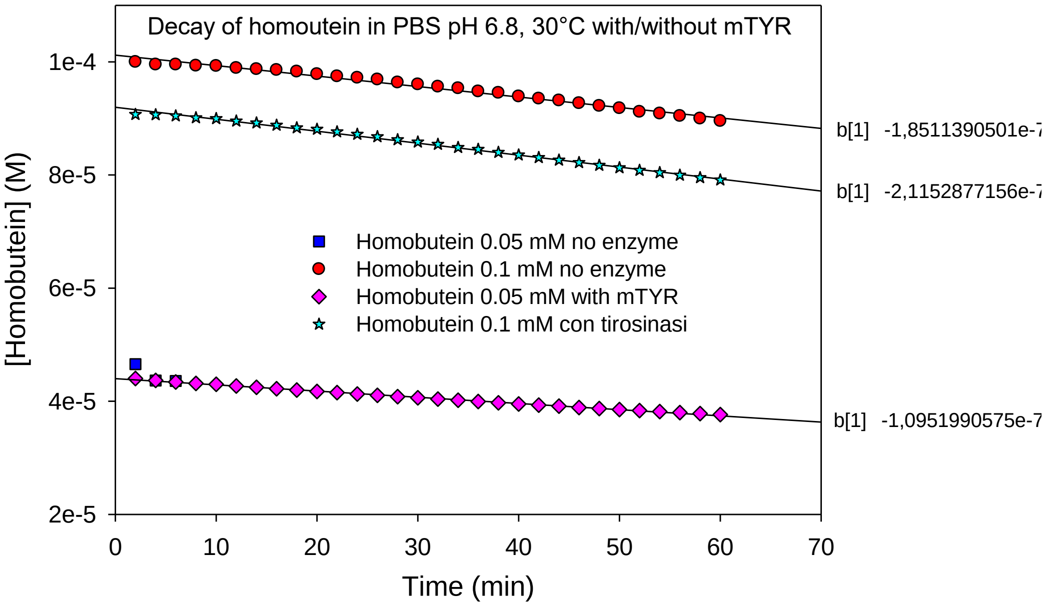

Figure S10. Kinetic analysis of the variation of homobutein concentration monitored at 385 nm in air-saturated PBS pH 6.8 at 30°C, in the presence and absence of mTYR, showing segmented zero-order fitting.

When butein (0.05 and 0.1 mM) incubated for 1h in air-saturated PBD at pH 6.8 (30°C) was added with 7 U/ml mTYR it showed rapid spectral variation with decay of the maximum at 390 nm and rapid growth of a signal at 361 followed by a slower decay of the signal at 390 and growth of a brad band at 485 nm (Figures S3, S4). Instead when it was incubated in the absence of enzyme the initial rapid spectral variation was not observed, but it was still visible the slow decay at 290 nm with growth of a band at 485-500 nm corresponding to a visual darkening of the solution (Figure S5).

Under identical settings, homobutein (0.05 and 0.1 mM) incubated with the enzyme did not show the rapid initial variation but just a slow decay of the signal at λ_{max} 385 nm and the behavior appeared identical when it was incuabated in the absence of enzyme in air-saturated PBS.

Analysis of the kinetics at λ_{max} 390 nm for butein in the presence of mTYR showed a behavior not represented by first order or second order kinetics, instead it could be reproduced by splitting the decay in two segments (one rapid and one slow) both of apparent zero-order. Using the extinction coefficient of 25014 $M^{-1}cm^{-1}$ determined at 390 nm, analysis showed a decay of its concentration of apparent zero-order (eq.1)

$$d[butein]/dt = k \qquad (1)$$

with $k_1 = 4.93 \times 10^{-5}$ $Mmin^{-1}$ and $k_2 = 3.32 \times 10^{-7}$ $Mmin^{-1}$ for the first and second segments, respectively with initial [butein] = 0.05 mM. In the absence of enzyme, the apparent zero order decay had instead $k = 3.46 \times 10^{-7}$ $Mmin^{-1}$ which was identical to the second segment in the presence of enzyme. On doubling the concentration to [butein] = 0.1 mM both rate constants approximately doubled showing a first order dependence on the concentration of the substrate. We attributed the first rapid decay to the formation of a complex with the enzyme, without substrate transformation, while the second segment was attributed to slow oxidation of butein, which was identical in the presence and absence of the enzyme, showing that butein is not a significant substrate for mTYR.

Analysis for homobutein was simplified by the absence of the initial rapid decay. Zero-order fitting of the slow decay at λ_{max} 385 nm at which an extinction coefficient of 29388 $M^{-1}cm^{-1}$ was estimated, afforded $k = 1.9 \times 10^{-7}$ $Mmin^{-1}$ in the absence of the enzyme and $k = 2.1 \times 10^{-7}$ $Mmin^{-1}$ in the presence on mTYR, both with [homobutein] = 0.1 mM and the values were approximately halved on halving the concentration of homobutein, again showing a fist order dependence on the concentration of the substrate. Similarly, we suggest that homobutein is not a significant substrate for mTYR.

Monitoring oxygen consumption upon incubation of butein and homobutein with mTYR

To confirm our conclusions, we monitored the oxygen consumption upon incubation of butein an homobutein in air-saturated PBS (pH 6.8, 30°C) in the presence and absence of mTYR. This is based on the fact that substrate oxidation by mTYR requires oxygen as the compulsory oxidant, so any transformation of the substrate by the enzyme would be accompanied by a consumption of oxygen, while formation of non-covalent complexes with the enzyme causing spectra variation would not produce any oxygen consumption.

As can be seen in the following Figure S10 and S11, oxygen consumption was extremely modest both for butein and for homobutein and it was almost identical int the presence and absence of the enzyme. This confirms that neither butein nor homobutein are significant substrates for mTYR. Both undergo slow oxidation is solution at pH 6.8, however the process is very slow compared to the mTYR kinetics with natural substrates l-tyrosine and L-dopa in which butein and homobutein are used as inhibitors. Therefore, any significant interference inhibitors kinetic behavior caused by their air-oxidation can be excluded under our experimental settings.

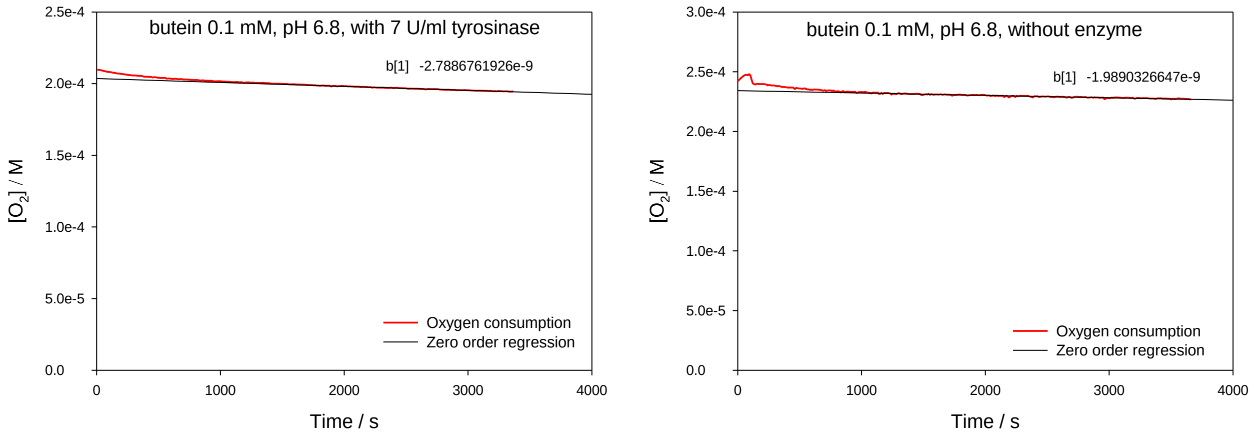

Figure S11. Oxygen consumption upon incubation of butein 0.1 mM in air-saturated PBS pH 6.8 at 30°C in the presence of 7U/mL mushroom tyrosinase (left) or in the absence of enzyme (right)

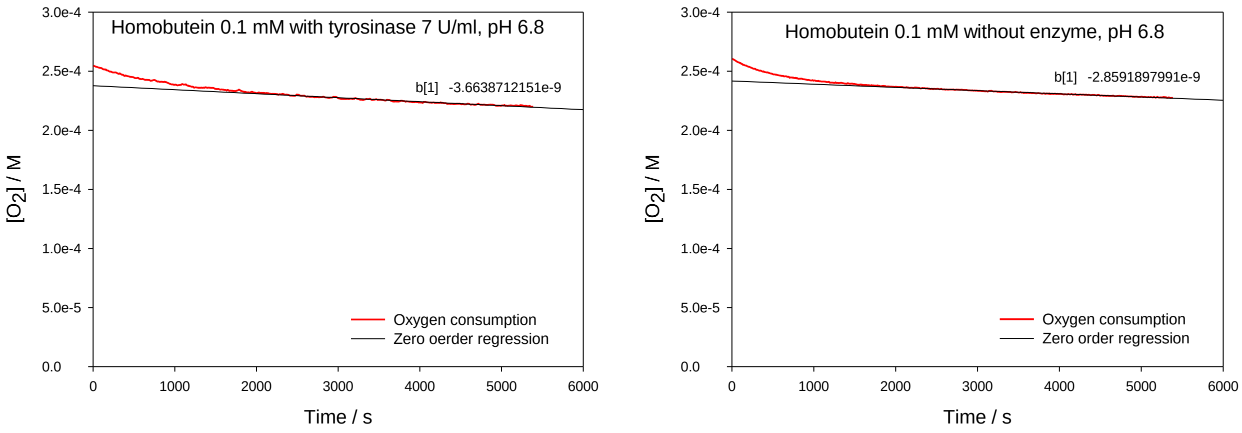

Figure S12. Oxygen consumption upon incubation of homobutein 0.1 mM in air-saturated PBS pH 6.8 at 30°C in the presence of 7U/mL mushroom tyrosinase (left) or in the absence of enzyme (right)
